# Knowledge and Perceived Readiness to Use Transcutaneous Electrical Nerve Stimulation for Primary Dysmenorrhea Among Female Undergraduates of the University of Ibadan

**DOI:** 10.1155/prm/9430375

**Published:** 2026-03-26

**Authors:** Iyanuoluwa Oreofe Ojo, Funmilayo Adenike Adegoroye, Ajibola Omobola Ojo, Olufemi O. Oyediran

**Affiliations:** ^1^ Department of Medical Surgical Nursing, Faculty of Nursing, University of Ibadan, Ibadan, Oyo State, Nigeria, ui.edu.ng; ^2^ Department of Maternal and Child Health Nursing, Faculty of Nursing, University of Ibadan, Ibadan, Oyo State, Nigeria, ui.edu.ng; ^3^ Department of Nursing, Obafemi Awolowo University, Ile Ife, Osun State, Nigeria, oauife.edu.ng

## Abstract

**Background:**

Primary dysmenorrhea (PD) is a gynecological disorder that affects the quality of life and academic performance of women of reproductive age. It is managed with oral nonsteroidal anti‐inflammatory medications, which may result in side effects that could be long term. Transcutaneous electrical nerve stimulation (TENS) is a noninvasive and cost‐effective procedure that does not require drugs in pain management, but there are low awareness and limited use of TENS among young women. This study aimed to assess the knowledge and perceived readiness to use TENS for PD among female undergraduates of the University of Ibadan.

**Method:**

A descriptive cross‐sectional study was conducted with a structured self‐administered questionnaire to gather data from 289 females with the use of a multistage sampling technique. The questionnaire was used to measure respondents’ knowledge of TENS, perceived readiness to use TENS, and barriers to its utilization, along with sociodemographic characteristics. The data were analyzed using IBM SPSS Version 25.0, using descriptive, bivariate, and multivariate statistics at a 0.05 significance level.

**Result:**

The mean age of the respondents was 22.4 ± 2.6 years. More than half of the respondents (79.9%) had poor knowledge of TENS, although over half (59.9%) of respondents were willing to use TENS for PD management. The main factors inhibiting the use of TENS were cost (67.1%) and limited awareness (68.5%). Logistic regression analysis showed that respondents with a monthly allowance of ₦5000–₦49,000 were 0.362 times less likely to have positive readiness for TENS. In contrast, the odds of positive readiness of TENS were 3.246 times higher among Adult Education female students (OR = 3.246, *p* = 0.020) and 2.693 times higher among Economics female students (OR = 2.693, *p* = 0.038) when compared to students of Veterinary Medicine.

**Conclusion:**

This study showed that most of the respondents had poor knowledge about the use of TENS. However, most of the participants were willing to use it. Limited awareness, financial constraints, and accessibility issues were identified as major barriers to the utilization of TENS. It is therefore important to enhance health education and digital literacy among female undergraduate students to encourage them to use online sources to learn about innovative, nonpharmacological options such as TENS for improving reproductive health and overall well‐being.

## 1. Introduction

Primary dysmenorrhea (PD) is a cramp‐pain​ that is experienced during menstruation in which the underlying cause is not known. It is a gynecological condition affecting more than half of women of reproductive age globally, with prevalence rates of about 90% among young women [1, 2]. The prevalence of this condition is widespread as a systematic review of the evidence in 38 countries showed that among young women, it occurs in an average of 71.1% [3]. Even though the severity of PD may differ, between 10% and 20% of cases are severe enough to interfere with daily routines, academic activities, and work productivity [1, 4]. Among the female population of the university students, the prevalence varies between 69.8% and 82.2% in Nigeria [1, 5–7], and a great number of students have claimed to have poor quality of life and absenteeism at school because of menstrual pain [5]. The significant prevalence of PD among university students has led to a growing focus on interventions that promote efficient pain management while reducing with academic life and daily functioning.

Pharmacological interventions such as nonsteroidal anti‐inflammatory drugs (NSAIDs) are widely used for PD management but may cause undesirable side effects, including gastrointestinal irritation, headaches, and renal complications [8]. This has led to a change in focus toward nonpharmacological approaches that are safer and more sustainable such as Transcutaneous Electrical Nerve Stimulation (TENS). It is a noninvasive, drug‐free type of pain relief, which utilizes low‐voltage electrical currents to prevent transmission of pain and induce an endorphin release, which is founded on the gate control theory of pain. Even though the analgesic effect is not permanent, TENS provides a drug‐free, low‐risk alternative for managing the pain associated with PD, and therefore, an intervention for patients seeking to avoid the side effects of prolonged medication use [9, 10].

Although TENS has shown itself to be clinically effective, research indicates that user’s knowledge, perceptions, and contextual factors also have an impact on utilization. In most developing countries, awareness and use of TENS for menstrual pain is still limited even though it is clinically effective with low risks. Cultural perceptions, high cost, and inadequate availability are some of the barriers that have been reported in the use of it in Tanzania [11]. However, such studies are lacking in Nigeria particularly in the southwestern part. It is necessary to note that the cultural and structural factors can be used to explain the influence TENS has on the acceptance and utilization by different populations. Building on this body of evidence, it is crucial to comprehend how potential users view and interact with TENS, especially in the university settings.

The importance of evaluating the knowledge and perceived readiness of female undergraduates to use TENS is important as this group constitutes a health‐conscious and well‐informed population that has the capability to impact larger menstrual health behavioral practices. Improved awareness of TENS will enable students to be able to better manage PD, decrease the use of analgesics, and improve academic performance and overall well‐being. Moreover, health‐related students who understand TENS may be better equipped to recommend nonpharmacological, evidence‐based interventions in their future professional practice.

Furthermore, improved knowledge can help nonhealth students manage their own dysmenorrhea, lessen their reliance on analgesics, and enhance their quality of life and academic performance. All things considered, the results of this study can direct university‐based menstrual health education programs, aid in clinical judgment, and encourage the incorporation of secure, easily accessible, and efficient pain management techniques into women’s health.

Therefore, this study aims to assess the knowledge, perceived readiness, and factors limiting the use of TENS for PD management among female undergraduate students at the University of Ibadan. It also examines the association between sociodemographic characteristics, such as monthly allowance and course of study, and knowledge of TENS.

## 2. Methodology

### 2.1. Research Design

A descriptive cross‐sectional design was used to assess the knowledge, perceived readiness, and barriers to the use of TENS for the management of PD. This design was chosen due to its suitability for collecting quantitative data from a large population within a defined period.

### 2.2. Study Participants

The study was carried out at the University of Ibadan, a Federal Tertiary Institution, consisting of sixteen faculties. The faculties include Clinical Sciences, Economics, Environmental Design and Management, Dentistry, Public Health, Basic Medical Sciences, Arts, Education, Veterinary Medicine, Science, Law, Pharmacy, Social Science, Renewable Natural Resources, Technology, and Agriculture.

Eight faculties were selected for inclusion in the study which was Education, Clinical Sciences, Agricultural Science, Technology, Veterinary Medicine, Economics, Pharmacy, and Science. These faculties were selected because they had a higher population of female students at the time of data collection. The study population comprised female undergraduate students from the selected faculties.

### 2.3. Sample Size and Sampling Technique

**FIGURE 1:**
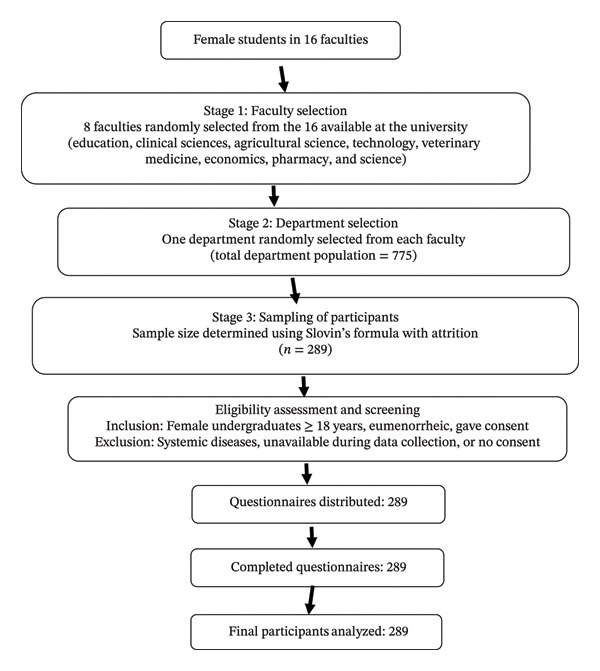
Flow diagram depicting the selection of study participants.

A multistage sampling technique was carried out. In the first stage, eight faculties were randomly selected from the sixteen faculties at the University of Ibadan. In the second stage, one department was randomly chosen from each of the eight selected faculties. The total population of female students across these selected departments was determined. Using the Slovin formula with an allowance for possible attrition, a final sample size of 289 participants was determined (Figure 1).

The cross‐sectional observational study employed exploratory logistic regression to identify factors associated with the study outcome; therefore, the sample size was determined primarily to ensure adequate population coverage rather than formal hypothesis testing power.

Participants were selected proportionately from the chosen departments to ensure fair representation across faculties. Only students who met the inclusion criteria were chosen to participate in the study.

### 2.4. Inclusion and Exclusion Criteria

The study included female students from the departments of adult education, nursing, pharmacy, agricultural extension, civil engineering, veterinary medicine, geology, and economics in their second, third, fourth, and fifth years of study.

The eligible participants were female undergraduate students aged 18 years and above who were eumenorrheic, willing to provide informed consent and had sufficient communication skills to comprehend and respond to the questionnaire.

Students who were unavailable during data collection, such as those on leave or vacation or who had systemic diseases that could affect menstrual patterns, were excluded from the study.

These exclusion criteria were used to reduce confounding variables and guarantee that participants could offer trustworthy data pertinent to the study’s goals. All initially chosen participants were screened for PD, as shown in the flow diagram above (Figure 1), and only those who satisfied the PD criteria were included in the final analysis.

### 2.5. Questionnaire Development

A self‐administered, structured 30‐item questionnaire was used to measure participants’ knowledge and perceived readiness and barriers to the use of TENS for PD management. The technology acceptance model (TAM), which holds that people’s adoption of a technology is primarily influenced by perceived usefulness and perceived ease of use, which in turn shape the intention to use and actual utilization, provided conceptual guidance for interpreting the questionnaire rather than serving as a formally tested model for this study. According to this framework, perceived readiness was thought to be preceded by knowledge of TENS, behavioral intention to use technology is conceptually reflected in perceived readiness, and perceived ease of use was considered influenced by identified barriers, although these constructs were not directly measured or empirically tested. The questionnaire was divided into four sections in which Section A captured sociodemographic characteristics (age, course of study, level, ethnic group, monthly allowance, and Internet access), Section B assessed knowledge of TENS, Section C measured perceived readiness to use TENS, and Section D examined barriers to its utilization, all using a “Yes/No” response format. Dichotomous scoring was employed to facilitate clarity and ease of response, particularly for knowledge‐based items, and to ensure straightforward classification of outcomes. While ordinal or Likert‐scale formats can capture response intensity, dichotomous scoring was considered fit for this exploratory analysis and study objectives.

The researchers created the questionnaire items, and five experts in adult health nursing and pain management assessed their face validity to ensure that they were clear, relevant, and appropriate.

There are 10 items in the knowledge section. The distribution of knowledge was based on 10 knowledge‐based questions with scores ranging from 0 to 10. The minimum score obtained by the female undergraduate was 0 and the maximum score was 10. The scores were categorized using modified Bloom’s cutoff in which scores below 6 (≥ 60%) were categorized as good knowledge, while scores < 6 (< 60%) were categorized as poor knowledge.

The perceived readiness section was assessed using 5 questions of yes/no type. Every Yes answer was given 1 point, and every No‐response received 0 points. The categorization was based on modified Bloom’s cut points in which ≥ 60% of the total obtainable score (≥ 3 points out of 5) was categorized as good readiness, while < 60% of the total obtainable score (< 3 points out of 5) was categorized as poor readiness.

### 2.6. Validation and Reliability of the Instrument

The questionnaire was evaluated through face validity by the five specialists in adult health nursing and pain management, who offered qualitative feedback on the relevance, clarity, simplicity, and cultural validity of each item. All items were rated on a 4‐point scale (1 = *not relevant* to 4 = *highly relevant)*. The Item‐Level Content Validity Indices (I‐CVI) were between 0.80 and 1.00, while the overall Scale‐Level Content Validity Index (S‐CVI/Ave) was found to be 0.93, indicating excellent content validity. The necessary changes to the content of the questionnaire were made based on their recommendations.

A test–retest was carried out over 2 weeks with 29 undergraduate students (10% of the study population) at another public tertiary institution to provide reliability. This pilot test was used to check the time spent filling in the questionnaire, understanding of wording, and sensitivity of barrier items. Cronbach’s alpha was at 0.745 which was confirmed to be acceptable as it established internal consistency of the instrument.

### 2.7. Data Collection Procedure

The data of the respondents were collected physically using the structured questionnaire administered by the researcher and two trained assistants. The questionnaires were distributed to consenting participants during lecture breaks and retrieved immediately after completion to ensure a high response rate. Participants were informed about the purpose of the study and assured of confidentiality. The average completion time for each questionnaire was approximately 15–20 min. Data were collected over a three‐month period, giving us enough time to reach participants and ensure thorough responses.

### 2.8. Method of Data Analysis

The data obtained for the study were cleaned and analyzed using IBM SPSS Statistics Version 25.0. Descriptive statistics, including frequencies and percentages, were used to summarize the data. Bivariate and multivariate analyses were performed to determine relationships between sociodemographic characteristics (monthly allowance and course of study) and the respondents’ knowledge and perceived readiness levels to use TENS. Statistical analysis was performed with a significant level of *p* ≤ 0.05. The sociodemographic characteristics (age, study level, course of study, monthly allowance, ethnicity, and Internet access) were the independent variables, and knowledge level and perceived readiness (good versus poor) were entered as dependent variables in separate models. Covariates were chosen based on data from the literature, and variables that showed strong bivariate correlations were taken into consideration when choosing covariates. The Hosmer–Lemeshow goodness‐of‐fit‐test was used to evaluate model fit after logistic regression assumptions were evaluated. This test verified that both models accurately represented the data. The study design guaranteed the independence of observations, and variance inflation factors were used to check for multicollinearity among predictor variables. Complete case analysis was used to exclude the few cases with missing data from the regression analyses.

### 2.9. Ethical Consideration

Ethical approval for this study was obtained from the University College Hospital and the University of Ibadan Ethical Research Committee (Approval No. UI/EC/24/0083). To ensure the anonymity and privacy of participants, personally identifiable information, such as names, addresses, and phone numbers, was not collected.

Participation in the study was voluntary and did not involve any invasive procedures, pain, or discomfort. Participants were informed that they could withdraw from the study at any time without penalty. All collected data were stored in a password‐protected Google Sheet, and physical questionnaires were shredded after data entry to ensure confidentiality. Participants were not compensated financially, but their time and contribution were acknowledged with verbal gratitude.

## 3. Results

### 3.1. The Sociodemographic Characteristics of Respondents

The result of sociodemographic characteristics of female undergraduates of the University of Ibadan is shown in Table 1. The respondents’ mean age was 22.4 ± 2.6 years. Most respondents, 205 (70.8%), were between the ages of 21 and 35 years, while 84 (29.1%) were within the 16–20 years age range. Slightly above half of the participants, 150 (51.9%), were in 300 level, 41 (14.2%) were in 200 level, 60 (20.8%) in 400 level, while 38 (13.1%) in 500 level.

**TABLE 1:** Sociodemographic characteristics of study participants (*N* = 289).

Variables	Categories	Frequency	Percentage
Age Mean ± SD: 22.4 ± 2.6	16–20	84	29.1
	21–25	205	70.8

Level of study	200	41	14.2
	300	150	51.9
	400	60	20.8
	500	38	13.1

Ethnic group	Yoruba	249	86.2
	Igbo	24	8.3
	Others	16	5.5

Monthly allowance (₦)	5000–50,000	248	85.8
	50,001–100,000	37	12.8
	100,001–150,000	4	1.4

Access to Internet	Yes	289	100

Course of study	Adult education	32	11.1
	Civil engineering	9	3.1
	Economics	38	13.1
	Geology	22	7.6
	Law	30	10.4
	Nursing	52	18.0
	Pharmacy	64	22.1
	Veterinary medicine	42	14.6

Most of the respondents, 249 (86.2%), were Yoruba, Igbo were 24 (8.3%), and other ethnic groups were 16 (5.5%). A large proportion of 248 (85.8%) respondents received a monthly allowance between ₦5000 and ₦50,000. All participants (100%) reported access to the Internet. The courses of study represented in the study in higher numbers were Pharmacy 64 (22.1%), Nursing 52 (18.0%), and Veterinary Medicine 42 (14.6%).

### 3.2. Knowledge of TENS

The participants’ knowledge of TENS was shown in Table 2. The results showed that less than one‐third (30.4%) of the participants knew about TENS and 15.2% of the participants got their knowledge about TENS from healthcare professionals. Less than one‐third (21.8%) obtained their knowledge from the Internet and social media, while only 6.2% of the participants got their knowledge from families and friends.

**TABLE 2:** Knowledge of transcutaneous electrical nerve stimulation machine.

Knowledge	Yes F (%)	No F (%)
Do you know about Transcutaneous Electrical Nerve Stimulation machine	88 (30.4)	201 (69.6)
Did you get the knowledge about TENS from a healthcare professional	44 (15.2)	245 (84.8)
Did you get the knowledge about TENS from the Internet and social media	63 (21.8)	226 (78.2)
Did you get the knowledge about TENS from families and friends	18 (6.2)	271 (93.8)
Do you know that TENS is a technology‐based painful menstruation management	82 (28.4)	207 (71.6)
Do you know that TENS is contraindicated in people with heart problems	35 (12.1)	254 (87.9)
Do you know that you can administer TENS machine by yourself	69 (23.9)	220 (76.1)
Do you know that TENS is applied on the surface of the skin	81 (28.0)	208 (72.0)
Do you know that the adverse effects of TENS is less compared to medications	0 (0.0)	289 (100.0)
Do you know that TENS is available in pharmacy stores and online stores	50 (17.3)	239 (82.7)

Regarding specific knowledge about TENS, less than one‐third (28.4%) of the participants knew that TENS is a technology‐based painful menstruation management technique. Less than one‐fifth (12.1%) were aware that TENS is contraindicated in people with heart problems. Less than one‐third (23.9%) knew that they could administer TENS machines themselves, and only 28.0% knew that TENS was applied on the surface of the skin. None of the participants knew that the adverse effects of TENS are less compared to medications. 17.3% of the respondents knew that TENS is available in pharmacy stores and online.

### 3.3. Distribution of Respondents According to Their Level of Knowledge of TENS

The level of knowledge of TENS among respondents is presented in Table 3. The total obtainable score was 10. The scores were categorized using a modified Bloom’s cutoff point in which scores ≥ 6 (≥ 60%) were categorized as good knowledge, while scores < 6 (< 60%) were categorized as poor knowledge. The results in this study showed that the majority, 231 (79.9%), had poor knowledge of TENS, while only 58 (20.1%) demonstrated good knowledge.

**TABLE 3:** Distribution of respondents according to their level of knowledge of TENS.

Level of knowledge	Category	Frequency	Percentage
Poor knowledge	0–5	231	79.9
Good knowledge	6–10	58	20.1
Total		289	100

### 3.4. Perceived Readiness to Use TENS for PD

The findings on participants perceived readiness to use TENS for PD are shown in Table 4. The results showed that none of the participants had ever used TENS; however, more than half, 182 (63.0%), showed willingness to use it if made available as an option. In the same way, more than half, 163 (56.4%), were willing to use TENS regardless of the severity of their menstrual cramps and a similar proportion 152 (52.6%) indicated preference for using TENS instead of analgesics such as felvin or paracetamol.

**TABLE 4:** Perceived readiness to use TENS for primary dysmenorrhea.

Perceived readiness	Yes	No
Do you use Transcutaneous Electrical Nerve Stimulation	0 (0.0)	289 (100.0)
If yes, are you willing to keep using TENS every time you have menstrual cramps	NA	NA
If no, are you willing to use TENS for your menstrual cramps if it is made available for you as an option	182 (63.0)	107 (37.0)
Are you willing to use TENS regardless of the severity of your menstrual cramps	163 (56.4)	126 (43.6)
Are you willing to use TENS for menstrual cramps instead of felvin or paracetamol	152 (52.6)	137 (47.4)

### 3.5. Distribution of Respondents by Level of Perceived Readiness of TENS

The distribution of respondents based on their perceived readiness to use TENS is shown in Table 5. The categorization was based on modified Bloom’s cut points in which ≥ 60% of the total obtainable score (≥ 3 points out of 5) was categorized as good perceived readiness, while < 60% of the total obtainable score (< 3 points out of 5) was categorized as poor perceived readiness. The results showed that more than half of the respondents, 173 (59.9%), showed good perceived readiness, while 116 (40.1%) demonstrated poor perceived readiness to use TENS.

**TABLE 5:** Distribution of respondents according to their level of perceived readiness of TENS.

Level of readiness	Categories	Frequency	Percentage
Poor	1‐2	116	40.1
Good	3–5	173	59.9
Total		289	100

### 3.6. Factors Inhibiting the Use of TENS Among University of Ibadan Female Students

**FIGURE 2:**
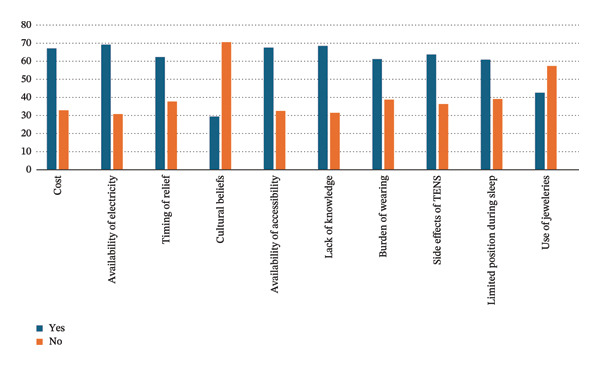
Factors inhibiting the use of TENS among the University of Ibadan female students.

The perceived barriers to the utilization of TENS are highlighted in Table 6 and shown in Figure 2. The results showed the most frequently reported barriers to be the availability of electricity (69.2%), limited awareness (68.5%), cost (67.1%), and timing of outcome measure relief after the application of TENS (62.3%). Other major factors inhibiting the use of TENS were the availability and accessibility of TENS in pharmacies (67.5%), the burden of wearing TENS during menstrual period identified by more than half (61.2%), and side effects of TENS reported by 63.7% of the respondents. Moreover, more than half (60.9%) of the participants identified limited position during sleep as a potential hindrance while less than half (42.6%) of the participants thought that wearing jewelry could hinder their use of TENS. Cultural beliefs about menstrual cramps were considered by less than one‐third (29.4%) as influencing the use of TENS.

**TABLE 6:** Factors inhibiting the use of TENS among the University of Ibadan female students.

Factors	Yes F (%)	No F (%)
Do you think the cost of TENS can affect your use of TENS	195 (67.1)	95 (32.9)
Do you think the availability of electricity can affect your use of TENS	200 (69.2)	89 (30.8)
Do you think timing of the outcome measure of relief after the application of TENS can affect your use of TENS	180 (62.3)	109 (37.7)
Do you think that cultural beliefs about menstrual cramps can affect your use of TENS	85 (29.4)	204 (70.6)
Do you think availability and accessibility of TENS in pharmacies can affect your use of TENS	195 (67.5)	94 (32.5)
Do you think your limited awareness about TENS can affect your use of TENS	198 (68.5)	91 (31.5)
Do you think wearing TENS during your menstrual period can be a burden to you	177 (61.2)	112 (38.8)
Do you think that the side effects of TENS can hinder you from using TENS	184 (63.7)	105 (36.3)
Do you think that limited position during sleep can hinder your use of TENS over the night	176 (60.9)	113 (39.1)
Do you think that your use of jewelries can hinder your use of TENS	123 (42.6)	166 (57.4)

### 3.7. Bivariate Analysis Between Sociodemographic Characteristics and Knowledge of TENS

The association between sociodemographic characteristics and knowledge of TENS among the University of Ibadan female undergraduates is shown in Table 7. The findings showed significant associations between knowledge of TENS and age (*p* = 0.007), level of study (*p* < 0.001), monthly allowance (*p* = 0.004), and course of study (*p* < 0.001). Ethnicity and Internet access showed no significant association with knowledge levels of TENS.

**TABLE 7:** Bivariate analysis between sociodemographic characteristics and knowledge of TENS.

Sociodemographic characteristics	Knowledge of TENS		*χ* ^2^	*p* value
	Poor *n* = 205 (%)	Good *n* = 84 (%)		
Age				
16–20	69 (23.9)	15 (5.2)	7.216	0.007^∗^
21–35	136 (47.1)	69 (23.9)		
Level of study				
200	30 (10.4)	11 (3.8)	26.258	0.001^∗^
300	121 (41.9)	29 (10.0)		
400	39 (13.5)	21 (7.3)		
500	15 (5.2)	23 (8.0)		
Ethnic group				
Yoruba	180 (62.3)	69 (23.9)	1.602	0.449
Igbo	15 (5.2)	9 (3.1)		
Others	10 (3.5)	6 (2.1)		
Monthly allowance (₦)				
5000–49,000	181 (62.6)	67 (23.2)	10.928	0.004^∗^
50,000–99,000	24 (8.3)	13 (4.5)		
100,000–150,000	0 (0.0)	4 (1.4)		
Access to Internet				
Yes	205 (70.9)	84 (29.1)		
Course of study				
Adult education	30 (10.4)	2 (0.7)	101.824	0.001^∗^
Civil engineering	8 (2.8)	1 (0.3)		
Economics	36 (12.5)	2 (0.7)		
Geology	21 (7.3)	1 (0.3)		
Law	25 (8.7)	5 (1.7)		
Nursing	9 (4.4)	43 (14.9)		
Pharmacy	44 (15.2)	20 (6.9)		
Veterinary medicine	32 (11.1)	10 (3.5)		

^∗^ *p* < 0.05 indicates statistical significance.

### 3.8. Multivariate Analysis Between Sociodemographic Characteristics and Knowledge of TENS

The results of the multivariate logistic regression (Table 8) showed that the level of study and course of study were significantly associated with knowledge of TENS. The results showed that when compared with 500‐level students, the odds of good knowledge of TENS were 5.14 times higher in 200‐level students (OR = 5.139, *p* = 0.023). Also, when compared with Veterinary Medicine female students, the odds of good knowledge of TENS were 10.51 times higher among adult education students (OR = 10.51, *p* = 0.009) and 8.89 times higher in Nursing students (OR = 8.89, *p* < 0.001), whereas Economics students had lower odds (OR = 0.103, *p* = 0.011).

**TABLE 8:** Multivariate analysis showing association between sociodemographic characteristics and knowledge of TENS.

Sociodemographic characteristics	B	Odd ratio	*p* value	95% CI
Age				
16–20	−0.135	0.874	0.767	0.358–2.132
21–35		1		
Level of study				
200	1.637	5.139	0.023	1.257–21.007
300	0.745	2.572	0.077	0.903–7.322
400	−0.061	0.941	0.916	0.303–2.921
500		1		
Monthly allowance (₦)				
5000–49,000	−21.166	0.642	0.346	0.642–1.439
50,000–99,000	0.166	1.181	0.725	0.468–2.982
100,000–150.000		1		
Course of study				
Adult education	2.352	10.510	0.009	1.815–60.852
Civil Engineering	1.580	4.854	0.175	0.495–47.634
Economics	−2.274	0.103	0.011	1.667–47.425
Geology	2.090	8.082	0.060	0.916–71.310
Law	1.328	3.774	0.059	0.952–14.961
Nursing	2.185	8.890	0.001	0.035–0.301
Pharmacy	0.168	1.183	0.740	0.438–3.189
Veterinary Medicine		1		

*Note:* *p* < 0.05 indicates statistical significance.

**FIGURE 3:**
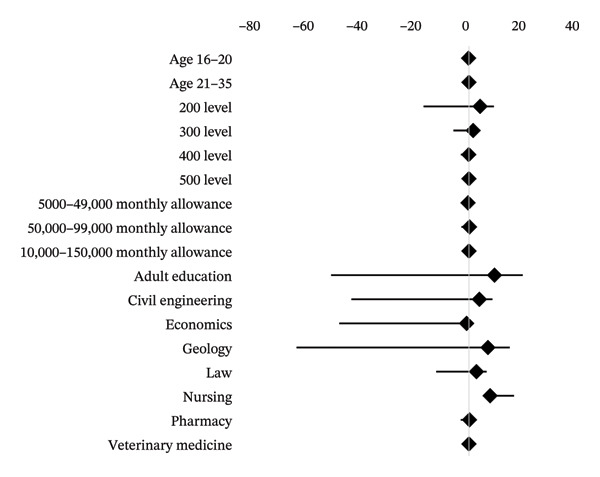
Multivariate logistic regression of sociodemographic characteristics associated with the knowledge of TENS among the University of Ibadan female undergraduates.

These associations can be seen in the multivariate logistic regression plot (Figure 3), where Adult Education and Nursing students have positive relationships and Economics students have lower odds compared to their Veterinary Medicine students.

### 3.9. Bivariate Analysis Between Sociodemographic Variables and Perceived Readiness of TENS

The perceived readiness of TENS was significantly associated with age (*p* = 0.029), monthly allowance (*p* < 0.001), and course of study (*p* < 0.001), as shown in Table 9. No significant associations were observed with ethnicity, level of study, or Internet access.

**TABLE 9:** Bivariate analysis showing association between sociodemographic variables and perceived readiness of TENS.

Sociodemographic characteristics	Readiness of TENS		*χ* ^2^	*p* value
Age				
16–20	42 (14.5)	42 (14.5)	4.793	0.029^∗^
21–35	74 (25.6)	131 (45.3)		
Level				
200	23 (8.0)	18 (6.2)	5.715	0.126
300	59 (20.4)	91 (31.5)		
400	20 (6.9)	40 (13.8)		
500	14 (4.8)	24 (8.3)		
Ethnic group				
Yoruba	95 (32.9)	154 (53.3)	3.022	0.221
Igbo	13 (4.5)	11 (3.8)		
Others	8 (2.8)	8 (2.8)		
Monthly allowance (₦)				
5000–49,000	110 (38.1)	138 (47.8)	13.329	0.001^∗^
50,000–99,000	6 (2.1)	31 (10.7)		
100,000–150,000	0 (0.0)	4 (1.4)		
Access to Internet				
Yes	116 (40.1)	173 (59.9)		
Course of study				
Adult education	20 (6.9)	12 (4.2)	35.345	0.001^∗^
Civil engineering	4 (1.4)	5 (1.7)		
Economics	23 (8.0)	15 (5.2)		
Geology	15 (9.2)	7 (2.4)		
Law	13 (4.5)	17 (5.9)		
Nursing	11 (3.8)	41 (14.2)		
Pharmacy	16 (5.5)	48 (16.6)		
Veterinary medicine	14 (4.8)	28 (9.7)		

^∗^ *p* < 0.05 indicates statistical significance.

### 3.10. Multivariate Analysis Between Sociodemographic Variables and Perceived Readiness of TENS

The results of multivariate analysis (Table 10) showed that monthly allowance and course of study were significantly associated with perceived readiness of TENS. It was shown that female students with monthly allowances of ₦5000–₦49,000 had odds of positive readiness of TENS that were 0.362 lower when compared to those earning a monthly allowance of ₦100,000–₦150,000 (OR = 0.362, *p* = 0.011). In contrast, the odds of positive readiness of TENS were 3.246 times higher among Adult Education female students (OR = 3.246, *p* = 0.020) and 2.693 times higher among Economics female students (OR = 2.693, *p* = 0.038) when compared to Veterinary Medicine students.

**TABLE 10:** Multivariate analysis showing association between sociodemographic variables and perceived readiness of TENS.

Sociodemographic characteristics	B	Odd ratio	*p* value	95% CI
Age				
16–20	0.296	1.344	0.318	0.762–2.402
21–35		1		
Monthly allowance (₦)				
5000–49,000	−19.435	0.362	0.011	0.321–1.124
50,000–99,000	−1.234	0.291	0.458	0.133–0.750
100,000–150,000		1		
Course of study				
Adult education	1.178	3.246	0.020	1.203–8.758
Civil engineering	0.531	1.701	0.494	0.372–7.785
Economics	0.990	2.693	0.038	1.056–6.863
Geology	1.357	3.885	0.192	1.254–12.030
Law	0.495	1.640	0.336	0.599–4.488
Nursing	−0.511	0.600	0.301	0.228–1.580
Pharmacy	−0.391	0.676	0.392	0.276–1.658
Veterinary medicine		1		

*Note:* *p* < 0.05 indicates statistical significance.

**FIGURE 4:**
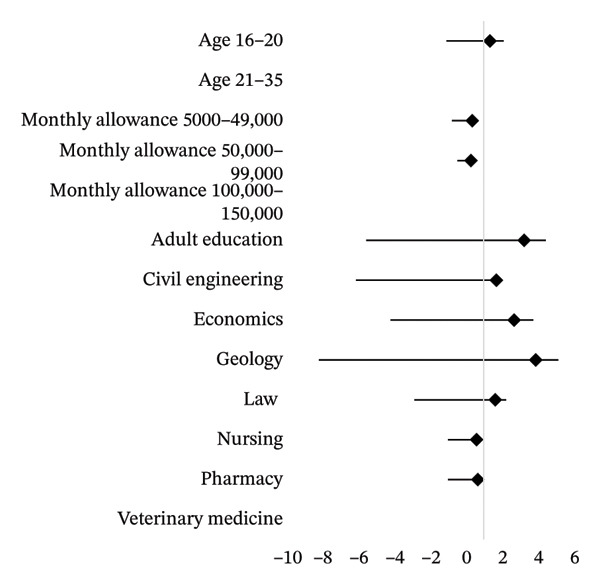
Multivariate logistic regression of sociodemographic characteristics associated with perceived readiness for TENS among the University of Ibadan female undergraduates.

The forest plot (Figure 4) showed the adjusted odds ratios of the perceived readiness and highlighted that the financial capability and academic discipline of the students are major factors in their readiness to use TENS.

## 4. Discussion

The management of PD has evolved over the years from traditional remedies and pharmacological therapies to modern nonpharmacological interventions such as TENS. Even though the use of TENS has been globally recognized as an effective, safe, and cost‐efficient alternative for menstrual pain relief [12, 13], its awareness and uptake remain limited among women of reproductive age in many developing countries, including Nigeria [14]. The findings from this study showed that the knowledge of TENS among female undergraduates was low with 29.1% of the respondents being aware of the device. The majority (73.4%) of the respondents expressed a positive perceived readiness to use it as an alternative to conventional analgesics. Cultural beliefs, affordability, and limited access were observed as major barriers to awareness and potential use of TENS.

### 4.1. Sociodemographic Characteristics

The average age of the respondents in this study was 23 years, with more than two‐thirds (68.5%) of the participants aged 21–30. This age distribution aligns with the findings of Ali et al. [12], who studied the prevalence, impact, and management perceptions of PD among university students and found that respondents had a similar age pattern between 18 and 30 years age range, implying a similar population structure for undergraduates. Unnisa et al. [15]​ also reported that most participants in their study of the assessment of the quality of life and nonpharmacological management of PD were between the age of 12–40 years, demonstrating that PD is frequently reported in a broad range of reproductive age group. The observed age range pattern is expected, given that the minimum university admission age in Nigeria is 16 years, and PD is most prevalent among women in their late teens and twenties [16]. This demographic is an important target population for menstrual health education and intervention, as early reproductive stages are generally characterized by a lack of awareness regarding nonpharmacological pain management techniques, which may influence their management decisions and health‐seeking behaviors.

### 4.2. Knowledge of the Use of TENS for PD

This study showed that the knowledge of TENS among female undergraduates in Ibadan was low (29.1%), with only about one‐third (18.7%) of the respondents aware of the device. This finding is in tandem with the study by Pietrzak et al. [17], who reported that awareness of nonpharmacological pain‐relief methods among women in Poland was low, with most of them being familiar with only basic techniques such as massage and breathing exercises, indicating a restricted spread of cutting‐edge pain treatment techniques outside of clinical settings. Similarly, Moradi et al. [18] reported in a clinical trial that although TENS was associated with reduced labor pain, many participants had no prior knowledge of the method before intervention; this supports the observation of poor baseline awareness among women prior to exposure by health providers.

Conversely, the awareness rate of TENS among the clinical community in Nigeria is extremely high. According to Mbada et al. [19], approximately 83% of the physiotherapists in Nigeria practiced transcutaneous electrical stimulation modalities, such as TENS, in daily care to manage pain. Another important aspect noted in the study was that physiotherapists in various practice environments utilize evidence‐based methods and show high professional awareness of therapeutic technologies. The significant difference between clinical awareness and low public knowledge noted during this study points to a significant gap in health communication and community education. To address this gap, there is a need to improve knowledge translation through targeted educational initiatives, curriculum integration, and sensitization effort focused on nonpharmacological pain management.

This study also discovered that nonmedical students, especially those in Economics (OR = 0.103, *p* = 0.011) and Adult Education faculties, demonstrated low levels of awareness compared to Nursing and Pharmacy students (OR = 8.890, *p* < 0.001). This difference was linked to differences in academic exposure, since health‐related disciplines are more likely to include pain management concepts and treatment technologies in their curricula. Therefore, adopting cross‐disciplinary education to promote health literacy may increase knowledge of evidence‐based treatments for menstruation discomfort.

Although earlier research has shown that TENS may help people with PD experience less discomfort [20], the current study did not evaluate its efficacy or clinical results. Instead, its results highlight knowledge gaps and disparities in female undergraduates’ perceptions of their preparedness to use TENS. Given the high prevalence of PD and its documented impact on productivity, these observations underscore the need for educational interventions and additional interventional research to examine the viability, acceptability, and efficacy of TENS within the Nigerian context [7].

### 4.3. Perceived Readiness to Use TENS for PD

Most respondents (73.4%) in this study indicated perceived readiness to use TENS for menstrual pain relief despite their limited knowledge of TENS. This finding is consistent with Unnisa et al. [15], who also observed that 70% of their participants were willing to use nonpharmacological options before resorting to analgesics. This is, however, at variance with Poncelas‐Cabero et al. [21], who found that cultural taboos and social learning patterns might cause women to choose self‐care practices or tolerate pain without resorting to medical treatment. In this study, the willingness of the participants to consider TENS, even though they were not familiar with it, suggested increasing openness among young women toward modern and nondrug pain management techniques.

This positive attitude may reflect the desire to avoid the side effects associated with prolonged use of NSAIDs, as highlighted by Ali et al. [12], who found that over 50% of female students relied primarily on medications for PD relief. The variations between these studies implies that, although the use of medication is still common, there is a growing interest in alternative management options such as TENS among young women.

### 4.4. Barriers to TENS Utilization

In this study, cultural beliefs and affordability were the primary challenges to the awareness and potential uptake of TENS. The respondents indicated that cultural beliefs (70.6%) were a barrier, which revealed a manifestation of enduring menstruation‐related stigma. Maulingin‐Gumbaketi et al. [22] also reported that secrecy and cultural taboos surrounding menstruation prevented menstrual health interventions in the Pacific Islands. In Nigeria and other parts of sub‐Saharan Africa, such taboos result in misinformation, shame, and reluctance to seek appropriate care [23]. These attitudes can contribute to the low awareness of emerging menstrual pain technologies such as TENS.

Economic barriers were also observed to be significant. Affordability and limited availability were noted to be key challenges among 67.1% and 67.5% of participants, respectively. These results were in line with the findings of Cherenack et al. [11], who emphasized that cost and accessibility were key factors that contributed to dysmenorrhea management in Tanzania. Access is further inhibited by high prices of medical devices in Nigeria, intermittent power supply, and the inaccessibility of these devices in the market. Subsidized programs, university‐based health initiatives, or local production are examples of exploratory solutions that may be used to solve the problem; their feasibility and acceptability could be investigated in subsequent research.

### 4.5. Bivariate and Multivariate Analysis Showing Associations Between Sociodemographic Characteristics and Knowledge of TENS

This study showed that sociodemographic variables, especially educational exposure, were significantly associated with knowledge of TENS. In the bivariate analysis, age was associated with knowledge (*χ* ^2^ = 7.216, *p* = 0.007), in which the older students (21–35 years) showed higher awareness of TENS. However, after controlling for other variables, age lost its independent significance in the multivariate model. This suggests that educational factors may be more strongly correlated to TENS knowledge than age differences alone. This is in line with the findings of Osei and Bjorklund [24], who emphasized that learning outcomes are primarily shaped by educational exposure and instructional context rather than age‐related differences, and Simon et al. [25], who demonstrated that the efficacy of TENS does not significantly differ across age groups, indicating that knowledge and familiarity are more influenced by experience and exposure than by chronological age.

The level of study and course of study were significant in both bivariate and multivariate analyses. The second‐year students were five times more likely to possess good knowledge than fifth‐year students (OR = 5.139, *p* = 0.023), due to early exposure through health awareness initiatives or peer learning. Similar results were reported by Meherali et al. [26], who found that early academic interventions improve health literacy among students.

The course of study was the most significant determinant of knowledge of TENS. Nursing and Pharmacy students showed nearly nine times greater odds of good knowledge (OR = 8.890, *p* < 0.001) when compared with Veterinary Medicine students, likely due to curriculum‐based exposure to pain management. Economics students, on the other hand, demonstrated significantly lower knowledge (OR = 0.103, *p* = 0.011), highlighting gaps in nonhealth disciplines and suggesting the need for health education initiatives to improve menstrual health awareness across all disciplines.

### 4.6. Bivariate and Multivariate Analyses Showing Associations Between Sociodemographic Variables and Perceived Readiness for TENS

The readiness to use TENS for PD management was associated with a combination of sociodemographic, economic, and educational factors. In the bivariate analysis, age, monthly allowance, and course of study showed significant associations with perceived readiness. Students aged 21–35 years were more willing to use TENS, an observation in this study supported by Damodar et al. [27], who reported that maturity and increased autonomy improve openness to health innovations. Monthly allowance also influenced perceived readiness, with higher perceived readiness seen among students earning ₦50,000–₦99,000, which is probably due to better financial capability to access the device.

Multivariate analysis also confirmed that the roles of economic capacity and academic discipline remained independently associated with perceived readiness. The students who stated that they received between ₦5000 and ₦49,000 earnings were significantly less likely to express readiness compared to students who received higher monthly earnings of between ₦100,000 and ₦150,000. This supports the study by Annevelink et al. [28], who stated that financial barriers can prevent access to new health technologies even when people are aware. After adjustment, the students in Adult Education (OR = 3.246, *p* = 0.020) and Economics (OR = 2.693, *p* = 0.038) show more readiness to use TENS than those in Veterinary Medicine. This suggested that the perceived readiness to use TENS is not completely determined by professional training but may also be a result of personal experiences with dysmenorrhea or intrinsic motivation to use nondrug alternatives. Kabir et al. [29] stated that personal health experiences can influence openness to new interventions beyond academic exposure.

Overall, this study suggests that a combination of knowledge, motivation, and financial considerations influences the perceived readiness to use TENS. These results demonstrate that despite limited awareness, a significant degree of perceived readiness was noted, suggesting that future educational and interventional research should prioritize overcoming financial and cultural barriers over immediate practice or policy recommendations.

## 5. Limitations of the Study

The quantitative cross‐sectional design of this study provided a broad overview but did not permit an in‐depth assessment of the participants understanding of the experiences and perceptions surrounding the use of TENS. It is possible that the use of dichotomous scoring oversimplified participant responses and made it more difficult to identify minute differences in knowledge and perceived readiness. To further evaluate the robustness of the results, sensitivity analysis and the use of ordinal or Likert‐scale metrics may be beneficial for future research. The study survey was also carried out in a single public higher institution in the Southwestern part of Nigeria, which might not reflect the different cultural and demographical contexts of the country. The lack of literature on TENS knowledge and utilization in PD in Nigeria also limited the possibility of comparing the results with other similar populations.

A multistage sampling technique was also used in the study, with a concentration on departments with a greater proportion of females. The sample might not accurately reflect the larger population of female undergraduates in Nigeria, even though participants were recruited proportionately to provide equitable representation across the specified faculties. Although baseline comparisons between departments and faculties showed internal consistency, their applicability to other colleges or areas is restricted.

Future studies should include participants from other regions, particularly the North and Southeast, where cultural inhibitions might have a greater influence on the adoption of TENS for dysmenorrhea. In addition, incorporating a mixed‐methods approach (both quantitative and qualitative) could provide a more comprehensive understanding of the topic.

## 6. Conclusion

This study showed that even though most female undergraduates demonstrated a positive willingness to use TENS for the management of PD, awareness and knowledge of the intervention were generally low. The major barriers included limited knowledge, high cost, and restricted access. Moreover, sociodemographic characteristics, particularly course of study and monthly allowance, had a considerable effect on knowledge and readiness to use TENS. The descriptive aspect of the study, which shows relationships rather than coincidental correlations, should be taken into consideration when interpreting these findings. Because the results are specific to the research population and setting, they are not applicable to all undergraduates or more general clinical situations. These results indicated that there is a need to implement specific educational interventions and enhance the availability of TENS as a safe, cost‐effective, and nonpharmacological approach to manage menstrual pain in young women.

However, these implications are intended to direct health education and awareness activities rather than provide precise recommendations for clinical care. More research in a range of settings is required to expand the body of evidence and promote wider implementation.

## 7. Implications for Health Education and Practice

The results highlight the need to incorporate menstrual health education in programs of tertiary institutions and more so to improve awareness nonpharmacological pain management methods such as TENS. Although TENS awareness was generally poor, students showed a high readiness to use the technology upon learning of its advantages, which indicates the potential value of targeted educational intervention rather than proven effect of an intervention. Evidence‐based lectures, practical demonstrations, and interactive workshops may help raise awareness and confidence of students in using TENS as well as preparedness to use it among those who had little previous exposure. Healthcare providers and educators should include alternate pain management strategies in the curriculum of reproductive health and campus health programs, focusing on instructional methods that equip students with theoretical understanding and practical skills rather than mandating implementation.

Students can be empowered by increasing awareness, affordability, and provision of user‐friendly instructional materials so that they can develop self‐managed and evidence‐based practices to dysmenorrhea that may help them decrease the use of analgesics and improve overall quality of life. Additionally, improving the interdisciplinary collaboration among physiotherapists, nurses, and health educators may help TENS become more widely known and accepted as a safe, scientifically supported method of managing menstruation pain. A combination of these educational strategies offers a model toward enhancing menstrual health literacy, reproductive health, and safe self‐care activities among female undergraduates.

## 8. Recommendation

Based on the findings of this study, it is recommended that female undergraduates, particularly those in nonhealth‐related disciplines, be supported to enhance their knowledge and perceived readiness to manage their reproductive health. Given their high access to the Internet, they should be encouraged to utilize online resources such as websites and blogs maintained by certified healthcare professionals to stay informed about conditions such as PD and nonpharmacological management options such as TENS. Although actual clinical use and patient outcomes were not evaluated, this strategy might assist close the noted knowledge gap and offer helpful, up‐to‐date health information. Targeted educational initiatives are important for improving the knowledge and perceived preparation regarding TENS for PD management. Such programs could be considered for incorporation into the school health curricula and public health efforts, but their effectiveness and feasibility would need to be evaluated in future studies. Adopting peer‐led educational initiatives may also help young women share their knowledge and promote perceived readiness in reliable social networks, without guaranteeing effectiveness. Additionally, comprehensive sexual and reproductive health education might be incorporated into college curricula, emphasizing TENS as a knowledge‐based alternative for PD management. This recommendation does not prove clinical efficacy or urgent policy necessity because it is based on self‐reported data. These suggestions act as hypotheses for upcoming interventional or longitudinal studies to investigate viability, acceptability, and possible health outcomes given Nigeria’s dearth to formal educational programs.

## Author Contributions

Study design: Iyanuoluwa Oreofe Ojo and Funmilayo Adenike Adegoroye.

Data collection: Funmilayo Adenike Adegoroye.

Data analysis: Iyanuoluwa Oreofe Ojo and Funmilayo Adenike Adegoroye.

Study supervision: Iyanuoluwa Oreofe Ojo, Funmilayo Adenike Adegoroye, and Ajibola Omobola Ojo.

Manuscript writing: Iyanuoluwa Oreofe Ojo, Funmilayo Adenike Adegoroye, Olufemi O. Oyediran, and Ajibola Omobola Ojo.

Critical revisions for important intellectual content: Iyanuoluwa Oreofe Ojo, Funmilayo Adenike Adegoroye, Olufemi O. Oyediran, and Ajibola Omobola Ojo.

## Funding

No funding was received for this research.

## Conflicts of Interest

The authors declare no conflicts of interest.

## Data Availability

The quantitative [numerical data] data used to support the findings of this study are available from the corresponding author upon request.

## References

[1] Esan D. T. , Ariyo S. A. , Akinlolu E. F. et al., Prevalence of Dysmenorrhea and Its Effect on the Quality of Life of Female Undergraduate Students in Nigeria, Journal of Endometriosis and Uterine Disorders. (2024) 5, 10.1016/j.jeud.2024.100059.

[2] Gebeyehu M. B. , Mekuria A. B. , Tefera Y. G. et al., Prevalence, Impact, and Management Practice of Dysmenorrhea Among University of Gondar Students, Northwestern Ethiopia: a Cross‐Sectional Study, International Journal of Reproductive Medicine. (2017) 2017, no. 1, 1–8, 10.1155/2017/3208276. PMC5446888 28589173

[3] MacGregor B. , Allaire C. , Bedaiwy M. A. , Yong P. J. , and Bougie O. , Disease Burden of Dysmenorrhea: Impact on Life Course Potential, International Journal of Women’s Health. (2023) 15, 499–509, 10.2147/IJWH.S380006. PMC10081671 37033122

[4] Itani R. , Soubra L. , Karout S. , Rahme D. , Karout L. , and Khojah H. M. J. , Primary Dysmenorrhea: Pathophysiology, Diagnosis, and Treatment Updates, Korean Journal of Family Medicine. (2022) 43, no. 2, 101–108, 10.4082/kjfm.21.0103. 35320895 PMC8943241

[5] Ezebialu I. U. , Ezenyeaku C. C. , and Umeobika J. C. , Prevalence of Dysmenorrhea and Its Contribution to School Absenteeism Among Nigerian Undergraduate Students. Annals of Health Research (The Journal of the Medical and Dental Consultants Association of Nigeria, OOUTH, Sagamu, Nigeria), Annals of Health Research. (2021) 7, no. 1, 59–66, 10.30442/ahr.0701-07-116.

[6] Ogunbamowo W. B. , Lafiaji-Okuneye B. B. , Ligali L. A. , and Ashon D. O. , Prevalence and Risk Factors of Dysmenorrhea Among Female Undergraduates in Lagos State University, International Journal of Human Kinetics, Health and Education. (2024) 9, no. 2.

[7] Okpata A. E. , Irene O. E. A. , Nwadinma N. J. , and Aribo Raneobhazi E. , Prevalence of Dysmenorrhea and Related Co-morbidities Among Adolescent Female Students in a Tertiary Institution in South South Nigeria, Saudi Journal Biomedical Research. (2024) 9, no. 6, 118–124, 10.36348/sjbr.2024.v09i06.003.

[8] Thakur P. and Pathania A. R. , Relief of dysmenorrhea–A Review of Different Types of Pharmacological and Non-Pharmacological Treatments, Materials Today: Proceedings. (2022) 48, 1157–1162, 10.1016/j.matpr.2021.08.207.

[9] Elboim-Gabyzon M. and Kalichman L. , Transcutaneous Electrical Nerve Stimulation (TENS) for Primary Dysmenorrhea: An Overview, International Journal of Women’s Health. (2020) 12, 1–10, 10.2147/IJWH.S220523. PMC6955615 32021488

[10] Melzack R. and Wall P. D. , Pain Mechanisms: a New Theory, Science. (1965) 150, no. 3699, 971–979, 10.1126/science.150.3699.971. 5320816

[11] Cherenack E. M. , Rubli J. , Melara A. et al., Adolescent Girls’ Descriptions of Dysmenorrhea and Barriers to Dysmenorrhea Management in Moshi, Tanzania: a Qualitative Study, PLOS Global Public Health. (2023) 3, no. 7, 10.1371/journal.pgph.0001544. PMC10325079 37410763

[12] Ali A. , Ali A. , Alotaibi N. S. et al., Prevalence, Impact, and Management Perception of Dysmenorrhea Among University Students: a cross-sectional Study, Brazilian Journal of Pharmaceutical Sciences. (2022) 58, 10.1590/s2175-97902022e20458.

[13] Chao T. , Zhang Y. , Liu H. , and Chen J. , Effectiveness of Transcutaneous Electrical Nerve Stimulation for Primary Dysmenorrhea: a Systematic Review and meta-analysis, Evidence-Based Complementary and Alternative Medicine. (2021) 2021, 10.1155/2021/6632326.

[14] Adeyemi A. S. , Olaniyi O. K. , and Afolabi O. T. , Awareness and Use of Transcutaneous Electrical Nerve Stimulation Among Women of Reproductive Age in Nigeria, Nigerian Medical Journal. (2023) 64, no. 3, 204–211, 10.60787/nmj-v64i3-259.

[15] Unnisa H. , Annam P. , Gubba N. C. , Begum A. , and Thatikonda K. , Assessment of Quality of Life and Effect of Non-Pharmacological Management in Dysmenorrhea. Annals of Medicine and Surgery, 2022, 81, 10.1016/j.amsu.2022.104407. PMC9486665 36147090

[16] Fagbamigbe A. F. , Obiyan M. O. , and Fawole O. I. , Parametric Survival Analysis of Menarche Onset Timing Among Nigerian Girls, Heliyon. (2018) 4, no. 12, 10.1016/j.heliyon.2018.e01105, 2-s2.0-85059115884. PMC6310774 30603722

[17] Pietrzak J. , Mędrzycka-Dąbrowska W. , Wróbel A. , and Grzybowska M. E. , Women′s Knowledge About Pharmacological and Non-pharmacological Methods of Pain Relief in Labor, Healthcare (Basel, Switzerland). (2023) 11, no. 13, 10.3390/healthcare11131882. PMC10340670 37444716

[18] Moradi F. , Alidousti K. , Zarbaf A. , Ghazanfarpour M. , Hosseinnataj A. , and Ahmadi A. , The Efficacy of Trans-cutaneous Electrical Nerve Stimulation (TENS) with Psychological Supportive Intervention on Pain and maternal-neonatal Outcomes of Childbirth in Pregnant Women Referring to Maternity Ward, The Iranian Journal of Obstetrics, Gynecology and Infertility. (2022) 25, no. 6, 61–70.

[19] Mbada C. E. , Onigbinde O. A. , Oyewole O. O. et al., Professional Practice Profile, Treatment Preferences, and the Bases for Clinical, Educational, and Research Among Nigerian Physiotherapists, Bulletin of Faculty of Physical Therapy. (2023) 28, no. 1, 10.1186/s43161-023-00159-2.

[20] Sanjeevi R. R. , Balasubramanian K. , AlShahrani M. N. et al., Effectiveness of self-administered High Frequency Pocket TENS in Pain Intensity, and Workability Among University Students with Primary Dysmenorrhea–A quasi-experimental Study Design, International Journal of Physical Therapy Research & Practice. (2025) 4, no. 4, 230–236, 10.62464/ijoprp.v4i4.101.

[21] Poncelas-Cabero P. M. , Andina-Díaz E. , Leirós-Rodríguez R. , Serrano-Gemes G. , and Rodríguez-Nogueira Ó. , Experiences and Perceptions of Primary Dysmenorrhea Mediating Management Behaviors: a Systematic Review of Qualitative Evidence and meta-aggregation, European Journal of Obstetrics & Gynecology and Reproductive Biology. (2025) 312, 10.1016/j.ejogrb.2025.114094. 40513262

[22] Maulingin-Gumbaketi E. , Larkins S. , Whittaker M. , Rembeck G. , Gunnarsson R. , and Redman-MacLaren M. , Socio-Cultural Implications for Women’s Menstrual Health in the Pacific Island Countries and Territories (PICTs): a Scoping Review, Reproductive Health. (2022) 19, no. 1, 10.1186/s12978-022-01398-7. PMC9164467 35655221

[23] Afolayan J. A. , Afolayan A. A. , Adejumo P. O. , and Olowookere A. S. , Menstrual Health Stigma, Misinformation and Care-Seeking Behaviour Among Women in sub-Saharan Africa: A Cross-sectional Study, African Health Sciences. (2023) 23, no. 2, 128–139, 10.4314/ahs.v23i2.14.

[24] Osei P. C. and Bjorklund D. F. , Motivating the Learning Process: Integrating self-determination Theory into a Dynamical Systems Framework, Educational Psychology Review. (2024) 36, no. 3, 10.1007/s10648-024-09934-6.

[25] Simon C. B. , Riley J. L. , Fillingim R. B. , Bishop M. D. , and George S. Z. , Age Group Comparisons of TENS Response Among Individuals with Chronic Axial Low Back Pain, The Journal of Pain. (2015) 16, no. 12, 1268–1279, 10.1016/j.jpain.2015.08.009, 2-s2.0-84949971997. 26342650 PMC4666741

[26] Meherali S. , Punjani N. S. , and Mevawala A. , Health Literacy Interventions to Improve Health Outcomes in Low- and Middle-Income Countries, Health Literacy Research and Practice. (2020) 4, no. 4, e251–e266, 10.3928/24748307-20201118-01. 33313935 PMC7751448

[27] Damodar P. , Shetty A. , Prakash A. , and Dsouza K. J. , Transformative Career Maturity Training for Rural Adolescents: an Exploratory Approach, International Journal of Adolescence and Youth. (2024) 29, no. 1, 10.1080/02673843.2024.2398044.

[28] Annevelink R. , Don S. , Nijs P. J. et al., Socio-Cultural Associates of Pain, Disability and health-related Quality of Life in 1,350 Primary Care Physiotherapy Patients with Non-specific Musculoskeletal Pain, Physiotherapy. (2025) 130, 10.1016/j.physio.2025.101804. 40835515

[29] Kabir H. , Hasan M. K. , and Mitra D. K. , E-learning Readiness and Perceived Stress Among the University Students of Bangladesh During COVID-19: a Countrywide cross-sectional Study, Annals of Medicine. (2021) 53, no. 1, 2305–2314, 10.1080/07853890.2021.2009908. 34889699 PMC8667940

